# Metabolic Control of Membrane Lipid Asymmetry in Cancer

**DOI:** 10.3390/ijms27093846

**Published:** 2026-04-26

**Authors:** Kyung-Hee Kim, Byong Chul Yoo

**Affiliations:** 1 Department of Applied Chemistry, School of Science and Technology, Kookmin University, Seoul 02707, Republic of Korea; kyungheekim@kookmin.ac.kr; 2 Antibody Research Institute, Kookmin University, Seoul 02707, Republic of Korea; 3 Diagnostic Research Team, InnoBation Bio R&D Center, Seoul 03929, Republic of Korea

**Keywords:** membrane lipid asymmetry, cancer metabolism, phospholipid redistribution, metabolic reprogramming, circulating metabolites, membrane remodeling, tumor microenvironment, membrane-targeted therapy

## Abstract

The plasma membrane plays essential roles in cellular transport and signaling. One of its fundamental structural features is the asymmetric distribution of lipids between the inner and outer leaflets. This asymmetry is actively maintained by lipid transport systems, including flippases, floppases, and scramblases, and is critical for membrane integrity and signaling regulation. Accumulating evidence indicates that membrane lipid asymmetry is frequently altered in cancer cells, leading to the externalization of normally inner-leaflet phospholipids such as phosphatidylserine and phosphatidylethanolamine. These alterations can influence tumor signaling, immune interactions, and membrane-associated biological processes. Recent studies further suggest that metabolic reprogramming in cancer may play an important role in regulating membrane lipid asymmetry. Changes in cellular energy status, oxidative stress, calcium signaling, and lipid metabolism can modulate lipid transport systems and membrane organization. In addition, tumor metabolism generates diverse circulating metabolites, including lactate, lysophospholipids, and acylcarnitines, which may influence membrane properties and lipid redistribution. These observations raise the possibility that membrane lipid asymmetry functions as a metabolically responsive interface linking intracellular metabolic state to cell surface signaling and tumor–microenvironment interactions. In this review, we propose a conceptual framework in which cancer-associated metabolic reprogramming influences lipid transport systems and membrane organization, thereby reshaping phospholipid distribution across the plasma membrane. We discuss how metabolic perturbations—including changes in energy metabolism, redox balance, calcium signaling, and lipid remodeling—may regulate membrane lipid asymmetry and explore the implications of these processes for tumor signaling, immune interactions, and emerging membrane-targeted therapeutic strategies.

## 1. Introduction

The plasma membrane is a dynamic and highly organized structure that plays central roles in cellular communication, signal transduction, and environmental sensing. Beyond serving as a physical barrier, the membrane functions as a regulatory interface that integrates metabolic state, signaling pathways, and extracellular interactions. One of the fundamental structural features of the plasma membrane is its lipid asymmetry, in which distinct lipid species are unevenly distributed between the inner and outer leaflets of the bilayer [1,2,3].

In most eukaryotic cells, phosphatidylcholine (PC) and sphingomyelin are predominantly located in the outer leaflet, whereas phosphatidylserine (PS), phosphatidylethanolamine (PE), and phosphatidylinositol (PI) are enriched in the inner leaflet of the plasma membrane [4,5]. This asymmetric distribution is not a passive property of membranes but is actively maintained by ATP-dependent lipid transporters, including flippases, floppases, and scramblases, which continuously regulate lipid translocation across the bilayer [6,7,8,9,10]. Maintenance of this lipid asymmetry is essential for membrane integrity, protein localization, and signaling processes.

Disruption of membrane lipid asymmetry has profound biological consequences. One of the most well-known examples is the externalization of PS, which occurs during apoptosis and serves as a recognition signal for phagocytic clearance of dying cells [11,12,13,14,15]. However, growing evidence indicates that lipid asymmetry can also be altered in a variety of non-apoptotic contexts, including inflammation, immune responses, coagulation, and tumor progression [16,17]. In particular, cancer cells frequently display abnormal exposure of inner-leaflet phospholipids such as PS and PE on the cell surface, a phenomenon that has been associated with tumor immune evasion, altered signaling networks, and therapeutic targeting opportunities [18,19,20,21,22,23].

While the molecular machinery that maintains lipid asymmetry has been studied for decades, a key question remains insufficiently explored: How does cellular metabolism influence membrane lipid asymmetry? Cancer cells undergo extensive metabolic reprogramming, including enhanced glycolysis, altered mitochondrial activity, and rewired lipid metabolism [24,25,26,27,28]. These metabolic alterations generate profound changes in intracellular energy status, redox balance, ion homeostasis, and lipid biosynthesis, all of which can potentially affect the activity of lipid-transporting enzymes and membrane organization. More recent work also suggests that metabolic plasticity contributes not only to tumor growth but also to therapy resistance, reinforcing the importance of understanding membrane remodeling within the broader context of cancer metabolic adaptation [29,30].

Recent studies suggest that metabolic conditions such as ATP depletion, oxidative stress, and calcium influx can directly modulate the activity of lipid transporters and scramblases, thereby altering membrane lipid distribution [31,32,33,34,35]. In addition, metabolic intermediates and circulating metabolites—including lactate, acylcarnitines, lysophospholipids, and other bioactive molecules—may influence membrane structure, lipid packing, and signaling pathways associated with lipid exposure [36,37,38,39,40,41]. These observations raise the possibility that membrane lipid asymmetry functions as a metabolic sensor, linking cellular metabolic state to membrane signaling and cell–environment interactions.

Understanding the metabolic regulation of membrane lipid asymmetry is particularly important in the context of cancer. Tumor cells exist in metabolically heterogeneous environments characterized by hypoxia, nutrient limitation, and altered metabolite exchange with the tumor microenvironment [42,43,44]. These conditions may promote membrane remodeling and lipid redistribution, thereby influencing processes such as immune recognition, EV formation, and therapeutic vulnerability [45,46,47]. From a translational perspective, abnormal surface exposure of PS has attracted increasing interest as both a biomarker and a therapeutic target, with preclinical and clinical studies supporting the feasibility of PS-directed imaging and treatment strategies [48,49,50,51].

At the same time, the consequences of altered lipid asymmetry must be considered within the broader framework of membrane organization. Membrane biophysical properties, including lipid packing, curvature, and raft organization, strongly influence protein recruitment and signaling output [52,53,54,55,56,57]. Thus, metabolic alterations that reshape membrane lipid composition may have downstream effects not only on lipid exposure itself but also on the functional architecture of the cancer cell surface.

In this review, we examine the emerging concept that membrane lipid asymmetry functions as a metabolically responsive interface linking intracellular metabolic state with cell surface signaling and tumor–microenvironment interactions. Although the enzymatic machinery responsible for maintaining lipid asymmetry has been extensively studied, considerably less attention has been given to how metabolic conditions regulate these systems and reshape membrane organization in cancer. Here we propose a conceptual framework connecting cancer metabolism, lipid transport systems, and membrane lipid asymmetry. Metabolic perturbations—including altered energy metabolism, redox imbalance, calcium signaling, and lipid remodeling—can influence the activity of flippases, floppases, and scramblases, thereby modulating phospholipid distribution across the plasma membrane. These metabolically driven membrane changes may in turn affect tumor signaling, immune interactions, extracellular vesicle (EV) formation, and therapeutic vulnerability.

**Figure 1:**
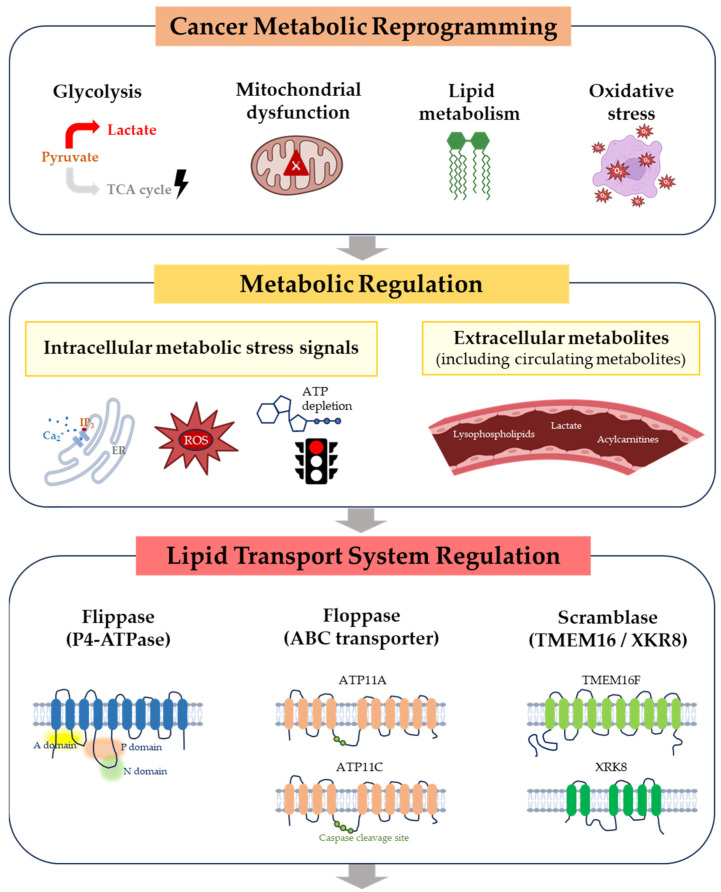

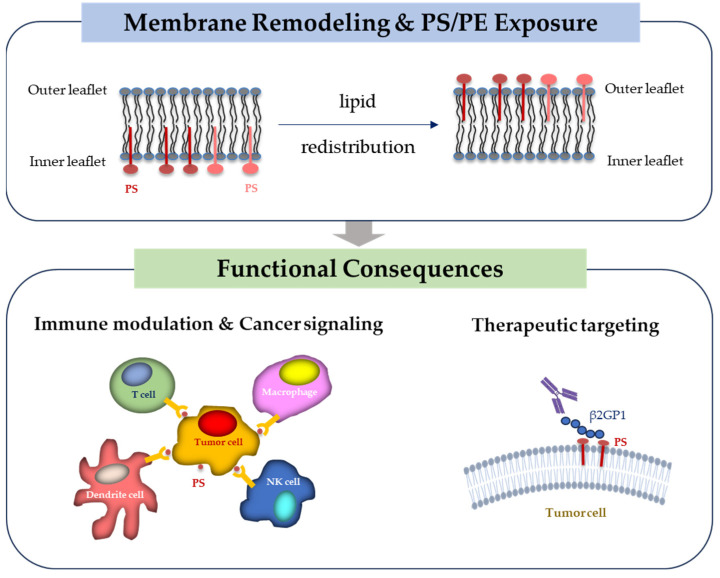
Conceptual framework linking cancer metabolism to membrane lipid asymmetry. Cancer metabolic reprogramming generates intracellular metabolic stress signals, including ATP depletion, oxidative stress, calcium signaling, and lipid remodeling. In parallel, extracellular metabolites (including circulating metabolites) present in the tumor microenvironment may also influence membrane properties and lipid transport systems. These metabolic inputs can modulate lipid transport systems such as flippases, floppases, and scramblases that regulate phospholipid distribution across the plasma membrane. Dysregulation of these transport mechanisms leads to redistribution of phospholipids between membrane leaflets and exposure of normally inner-leaflet lipids such as PS and PE on the cell surface. Altered membrane lipid asymmetry can subsequently influence tumor signaling, tumor–immune interactions, EV formation, and membrane-targeted therapeutic strategies.

Accordingly, we first summarize the molecular mechanisms responsible for maintaining lipid asymmetry in biological membranes. We then discuss how metabolic pathways and metabolic stress influence lipid transport systems and membrane organization. Finally, we explore the implications of metabolically controlled lipid asymmetry for cancer signaling, tumor–immune interactions, and emerging membrane-targeted therapeutic strategies (Figure 1).

## 2. Molecular Basis of Membrane Lipid Asymmetry

Biological membranes exhibit a highly organized lipid architecture in which specific lipid classes are asymmetrically distributed between the inner and outer leaflets of the bilayer. In the plasma membrane of mammalian cells, PC and sphingomyelin are primarily enriched in the outer leaflet, whereas PS, PE, and PI species are predominantly localized to the cytoplasmic leaflet [4,5,8]. This asymmetric lipid distribution is essential for membrane curvature, electrostatic interactions with cytosolic proteins, and the spatial organization of signaling complexes [52,53,54,55,56].

Maintenance of lipid asymmetry is an energy-dependent process controlled by several classes of lipid transporters. The major lipid transport systems involved in maintaining membrane lipid asymmetry are summarized in Table 1. Flippases, which belong mainly to the P4-ATPase family, transport aminophospholipids such as PS and PE from the outer leaflet to the inner leaflet using ATP hydrolysis [6,7,10]. In contrast, floppases, typically members of the ATP-binding cassette (ABC) transporter family, move lipids from the inner leaflet toward the extracellular leaflet [6].

A third class of transport proteins, known as scramblases, mediates bidirectional and ATP-independent lipid translocation across the membrane [31,58]. Unlike flippases and floppases, scramblases collapse lipid asymmetry by allowing phospholipids to equilibrate between the two leaflets. Several scramblases have been identified, including the TMEM16 and XKR protein families, which play critical roles in processes such as apoptosis, blood coagulation, and cell activation [31,32,34,35,59].

**Table 1:** Lipid Transport Systems Regulating Membrane Lipid Asymmetry.

Transport System	Representative Proteins	Transport Direction	Major Lipid Substrates	Biological Roles	Key Reference
Flippases	P4-ATPases (ATP11A, ATP11C, ATP8A1)	Outer → Inner leaflet	PS, PE	Maintenance of membrane lipid asymmetry; membrane charge regulation	[6,7,8]
Floppases	ABC transporters (ABCA1, ABCB1, ABCG1)	Inner → Outer leaflet	PC, cholesterol, phospholipids	Lipid export; membrane remodeling; lipid homeostasis	[2,6]
Scramblases (Ca^2+^-activated)	TMEM16 family (TMEM16F)	Bidirectional	PS, PE, phospholipids	Rapid lipid redistribution during cell activation	[31,32,34]
Scramblases (apoptosis-related)	XKR family (XKR8, XKR9)	Bidirectional	PS, PE	PS exposure during apoptosis; cell clearance signals	[13,16,59]
Other lipid transport regulators	CDC50 family proteins	Cofactors for P4-ATPases	PS, PE	Stabilization and activation of flippases; membrane regulation	[7]

The coordinated activities of these lipid transport systems ensure the dynamic maintenance of membrane asymmetry while allowing rapid remodeling when required by cellular signals. Disruption of this balance can lead to exposure of inner-leaflet phospholipids on the cell surface, altering membrane charge distribution and enabling new interactions with extracellular proteins, immune cells, and signaling molecules [9,11,15,16,20]. Consequently, lipid asymmetry represents not only a structural feature of biological membranes but also an important regulatory element in cellular physiology.

## 3. Alterations of Membrane Lipid Asymmetry in Cancer

Alterations in membrane lipid composition and distribution are increasingly recognized as hallmarks of cancer cell biology. In addition to genetic and metabolic reprogramming, tumor cells frequently undergo extensive remodeling of plasma membrane architecture. These changes affect not only the overall lipid composition of the membrane but also the asymmetric distribution of phospholipids between the inner and outer membrane leaflets. Disruption of lipid asymmetry can influence membrane charge, protein localization, and cell–environment interactions, thereby contributing to multiple aspects of tumor progression [3,28,60,61].

### 3.1. PS Exposure in Tumor Cells

Among the various alterations observed in tumor cell membranes, externalization of PS is one of the most extensively studied phenomena. Under normal physiological conditions, PS is almost exclusively confined to the cytoplasmic leaflet of the plasma membrane, where it contributes to membrane charge and supports the localization of numerous signaling proteins [4,5]. The maintenance of PS asymmetry is primarily mediated by ATP-dependent flippases that continuously transport PS from the outer leaflet back to the inner leaflet [6,7].

During apoptosis, lipid asymmetry collapses and PS becomes exposed on the cell surface, serving as an “eat-me” signal for phagocytic clearance [11,13,14,16]. However, numerous studies have shown that viable tumor cells can also display PS on their outer membrane surface without undergoing apoptosis [18,19,22]. This abnormal PS exposure has been documented in a wide variety of cancers, including breast cancer, melanoma, pancreatic cancer, and glioblastoma [19,20].

Surface-exposed PS can have important biological consequences in the tumor microenvironment. PS has been shown to promote immunosuppressive signaling, facilitating the recruitment of regulatory immune cells and suppressing anti-tumor immune responses [20,50]. In addition, PS exposure can influence interactions between tumor cells and endothelial cells, potentially contributing to tumor angiogenesis and metastasis [19,21].

Because PS exposure appears to be more prevalent in tumor cells than in normal tissues, it has attracted considerable interest as a therapeutic target. Several experimental therapies have been developed to exploit this property, including PS-binding antibodies, lipid-targeting peptides, and nanoparticle-based delivery systems designed to selectively recognize PS-exposing cells [48,49].

### 3.2. Exposure of PE and Other Inner-Leaflet Lipids

In addition to PS, other inner-leaflet phospholipids such as PE have also been reported to appear on the surface of tumor cells under certain conditions [21]. PE plays an important role in membrane curvature and bilayer stability, and its asymmetric distribution contributes to the mechanical properties of biological membranes [52,53]. While PE is often discussed alongside PS as an inner-leaflet phospholipid, it exhibits distinct biophysical properties that are highly relevant to membrane organization. Unlike cylindrical phospholipids such as PC, PE possesses a cone-shaped molecular geometry due to its relatively small headgroup and bulky acyl chains. This structural feature confers an intrinsic tendency to adopt non-bilayer configurations, particularly the inverted hexagonal (HII) phase, rather than a lamellar bilayer structure [62].

In biological membranes, however, PE is maintained within the bilayer through stabilizing interactions with other lipids, including PC, sphingomyelin, and cholesterol, as well as through protein–lipid interactions that collectively preserve membrane integrity [54,55]. The presence of PE within the bilayer contributes to membrane curvature, lateral pressure distribution, and packing stress, all of which are critical for dynamic membrane processes such as vesicle formation, membrane fusion, and protein conformational changes [52,53]. Importantly, the non-bilayer propensity of PE implies that its externalization is not merely a passive redistribution event but may reflect underlying alterations in membrane curvature and lipid packing. In the context of cancer, where metabolic stress, oxidative damage, and altered lipid composition are prevalent, changes in PE distribution may therefore be closely linked to membrane remodeling processes and the mechanical properties of the plasma membrane.

Exposure of PE on the cell surface can influence interactions with lipid-binding proteins and peptides. Certain antimicrobial peptides and synthetic membrane-active molecules preferentially interact with PE-rich membranes, suggesting that altered PE distribution may contribute to the increased susceptibility of tumor cells to membrane-disrupting agents [22,23].

Furthermore, exposure of inner-leaflet lipids may affect receptor signaling and membrane protein function. Because the cytoplasmic leaflet of the membrane normally carries a negative charge due to the presence of PS and other anionic phospholipids, redistribution of these lipids can alter electrostatic interactions that control protein recruitment and signal transduction [4,54].

### 3.3. Membrane Lipid Remodeling in Cancer

Beyond the redistribution of specific phospholipids, cancer cells often exhibit broader changes in membrane lipid composition. Tumor cells frequently display alterations in cholesterol levels, sphingolipid metabolism, and fatty acid saturation patterns [41,60,61,63]. These changes can influence membrane fluidity, lipid raft formation, and the spatial organization of membrane-associated signaling complexes.

Lipid rafts, which are cholesterol- and sphingolipid-rich membrane microdomains, play key roles in organizing signaling pathways involved in cell proliferation, migration, and immune interactions [57,64,65]. Alterations in lipid raft composition have been reported in several cancers and may influence the activation of oncogenic signaling pathways [66].

Changes in fatty acid saturation can also modify membrane properties. Increased levels of unsaturated fatty acids generally enhance membrane fluidity, whereas saturated lipids promote membrane rigidity and ordered lipid phases [67]. These properties can affect the mobility of membrane proteins and the accessibility of lipids to transport systems responsible for maintaining membrane asymmetry.

### 3.4. EVs and Lipid Redistribution

EVs, including exosomes and microvesicles, are increasingly recognized as important mediators of intercellular communication in cancer. Tumor cells release large numbers of EVs containing proteins, nucleic acids, and lipids that can influence the behavior of neighboring cells and distant tissues [45,46,47].

The formation of EVs is closely associated with changes in membrane lipid asymmetry. For example, outward budding of the plasma membrane during microvesicle formation often involves localized exposure of PS and redistribution of phospholipids between membrane leaflets [5]. Such lipid rearrangements can facilitate membrane curvature and vesicle scission [52].

EVs derived from tumor cells often display distinct lipid compositions compared with the parent cell membrane, including enrichment of certain phospholipids and sphingolipids [40,68,69]. These lipid changes may influence vesicle stability, targeting, and uptake by recipient cells.

### 3.5. Influence of the Tumor Microenvironment

The tumor microenvironment plays a significant role in shaping membrane lipid organization. Tumor cells are frequently exposed to conditions such as hypoxia, oxidative stress, and nutrient limitation, all of which can influence membrane remodeling processes [33,42,43].

Interactions between tumor cells and stromal cells, immune cells, and extracellular matrix components may also affect membrane lipid dynamics. Cytokines, growth factors, and extracellular metabolites present in the tumor microenvironment can activate signaling pathways that influence membrane trafficking, vesicle formation, and lipid metabolism [25,29,30,44].

Together, these factors contribute to a dynamic membrane landscape in which lipid asymmetry and membrane composition are continuously reshaped in response to environmental cues. Understanding these processes is essential for elucidating how tumor cells adapt their membrane architecture to support survival, communication, and immune evasion.

## 4. Metabolic Regulation of Lipid Transport Systems

Maintenance of membrane lipid asymmetry requires continuous and tightly regulated activity of lipid transport proteins. Because many of these transport processes depend directly or indirectly on cellular metabolic state, membrane lipid distribution is highly sensitive to metabolic perturbations. In cancer cells, where metabolic pathways are extensively rewired, alterations in energy metabolism, redox balance, ion signaling, and lipid biosynthesis may collectively influence the activity of lipid transport systems and thereby reshape membrane lipid asymmetry [24,25,26,28]. These observations suggest that membrane lipid asymmetry should not be viewed solely as a structural feature of the plasma membrane, but rather as a dynamic property that can respond to metabolic cues and cellular stress.

### 4.1. Energy Metabolism and ATP-Dependent Lipid Transport

Flippases responsible for maintaining aminophospholipid asymmetry belong primarily to the P4-ATPase family and require ATP hydrolysis to transport phospholipids from the outer to the inner leaflet of the plasma membrane [6,7,8,10]. Consequently, cellular ATP levels are critical determinants of flippase activity. Experimental depletion of intracellular ATP has been shown to inhibit flippase-mediated lipid transport, resulting in accumulation of PS and PE on the outer leaflet of the plasma membrane [5].

Cancer cells exhibit profound alterations in energy metabolism, including enhanced glycolysis, altered mitochondrial function, and fluctuating ATP production under hypoxic conditions [24,25,27]. Although cancer cells often maintain high glycolytic flux to sustain ATP generation, local ATP depletion can occur in metabolically stressed regions of tumors, such as hypoxic or nutrient-deprived microenvironments [42,43]. Under these conditions, impaired ATP-dependent lipid transport may contribute to partial loss of membrane lipid asymmetry. This apparent paradox can be partially explained by the spatial and temporal heterogeneity of energy metabolism in cancer cells. Although the Warburg effect supports sustained ATP production through enhanced glycolysis [24], ATP availability is not uniform across the cell. Localized ATP depletion may occur in regions with high energy demand, such as sites of active membrane remodeling, ion transport, or cytoskeletal reorganization. In addition, tumor microenvironments characterized by hypoxia and nutrient limitation can further constrain ATP supply [42,43]. Therefore, even in metabolically active cancer cells, local energy insufficiency may impair ATP-dependent lipid transport processes, contributing to dynamic and partial loss of membrane lipid asymmetry.

In addition, the spatial distribution of ATP within cells may influence local membrane remodeling. Recent studies suggest that membrane-associated ATP consumption by ion pumps, cytoskeletal remodeling, and vesicle trafficking can generate localized metabolic gradients that affect nearby membrane processes [44]. These local energy constraints may influence flippase activity and contribute to dynamic membrane lipid redistribution during processes such as migration, endocytosis, or EV formation.

### 4.2. Calcium Signaling and Scramblase Activation

Intracellular calcium signaling is another major regulator of membrane lipid asymmetry. Several scramblases belonging to the TMEM16 (anoctamin) family are activated by elevated intracellular calcium levels and can rapidly collapse lipid asymmetry by facilitating bidirectional phospholipid translocation across the membrane [31,32,34,35,58].

Activation of TMEM16 scramblases can lead to rapid externalization of PS and PE within seconds to minutes following calcium influx [31]. Such calcium-dependent lipid scrambling plays important roles in physiological processes including blood coagulation, platelet activation, and cell membrane repair [5,9].

Cancer cells frequently display altered calcium signaling due to dysregulation of ion channels, metabolic stress, and interactions with the tumor microenvironment [42,44]. Increased intracellular calcium levels can arise from hypoxia, oxidative stress, or activation of signaling pathways that mobilize calcium from intracellular stores [43]. These conditions may promote scramblase activation and contribute to the abnormal exposure of inner-leaflet phospholipids on the surface of tumor cells.

Furthermore, calcium signaling is closely linked to metabolic pathways. Mitochondrial metabolism, reactive oxygen species (ROS) production, and metabolic enzyme activity can influence calcium homeostasis, creating additional connections between metabolic state and membrane lipid redistribution [33].

### 4.3. Redox Regulation and Oxidative Stress

Redox balance represents another important link between metabolism and membrane lipid organization. Cancer cells often experience elevated levels of ROS due to increased mitochondrial activity, oncogenic signaling, and metabolic stress [33,70]. ROS can directly affect membrane structure by inducing lipid peroxidation, which alters membrane fluidity, curvature, and lipid packing [71].

Oxidative modification of membrane phospholipids can influence their interactions with membrane proteins and may increase their susceptibility to translocation across the bilayer [72,73]. In addition, oxidative stress has been reported to modulate the activity of lipid transporters and scramblases, although the precise mechanisms remain incompletely understood [33].

Lipid peroxidation products, including oxidized phospholipids and reactive aldehydes, can also act as signaling molecules that influence cellular stress responses and inflammation [72]. These processes may further contribute to membrane remodeling in cancer cells, particularly within hypoxic or metabolically stressed tumor regions.

### 4.4. Lipid Metabolism and Phospholipid Remodeling

Cancer cells undergo extensive alterations in lipid metabolism to support rapid proliferation and membrane biogenesis. Enhanced fatty acid synthesis, altered phospholipid biosynthesis, and increased lipid remodeling activity are hallmarks of cancer metabolism [41,60,61,63]. These metabolic changes can significantly influence the composition and biophysical properties of cellular membranes.

The relative abundance of specific phospholipid species affects membrane curvature, charge distribution, and interactions with membrane proteins. For example, PS contributes to the negative charge of the cytoplasmic leaflet, which facilitates the recruitment of signaling proteins containing polybasic domains [4]. Alterations in phospholipid composition may therefore modify the stability of lipid asymmetry and influence the accessibility of lipids to transport systems.

Phospholipid remodeling through the Lands cycle, in which acyl chains are removed and reattached by phospholipases and acyltransferases, can further modify membrane properties [74,75]. Changes in fatty acid saturation levels affect membrane fluidity and lipid packing, which may influence the efficiency of lipid transport across the bilayer. Cholesterol content also plays a critical role in membrane organization. Cholesterol-rich microdomains, often referred to as lipid rafts, contribute to membrane rigidity and compartmentalization of signaling proteins [57,64,65]. Alterations in cholesterol metabolism in cancer cells may therefore affect the dynamics of membrane lipid distribution and lipid–protein interactions.

### 4.5. Post-Translational Regulation of Lipid Transport Proteins

In addition to metabolic influences on membrane lipids themselves, the activity of lipid transport proteins can be regulated through post-translational modifications and signaling pathways. For example, certain scramblases are activated by proteolytic cleavage during apoptosis, particularly through caspase-mediated activation of XKR family proteins [31,59].

Although this mechanism is best characterized in apoptotic cells, partial activation of related pathways may occur in non-apoptotic contexts such as cellular stress or inflammatory signaling. Phosphorylation and other regulatory modifications may also influence the activity or localization of lipid transporters. Signaling pathways frequently altered in cancer, including kinase cascades and stress-response pathways, could therefore indirectly modulate membrane lipid asymmetry [25].

Taken together, these mechanisms suggest that membrane lipid asymmetry is not a static structural property but rather a dynamic feature that can respond to metabolic and signaling cues. In cancer cells, metabolic reprogramming may therefore influence membrane organization through multiple interconnected pathways, ultimately contributing to the altered lipid exposure patterns observed in tumors.

## 5. Circulating Metabolites and Membrane Remodeling

Cancer metabolism produces profound alterations not only in intracellular metabolic pathways but also in the composition of metabolites present in the tumor microenvironment and systemic circulation. Tumor cells release a wide variety of metabolic intermediates and bioactive molecules, including lactate, organic acids, and lipid metabolites. Examples of circulating metabolites potentially influencing membrane remodeling are summarized in Table 2. Although direct experimental evidence remains limited, emerging metabolomic observations suggest that circulating metabolites may contribute to membrane remodeling. Increasing evidence indicates that tumor-derived metabolites can influence cellular signaling, immune responses, and tissue remodeling [24,25,26,29,30]. Whether such metabolites directly regulate membrane lipid asymmetry remains largely unexplored, representing an important area for future investigation.

In addition, their potential role in regulating membrane lipid organization and asymmetry has only recently begun to attract attention. Because biological membranes are highly sensitive to the physicochemical properties of surrounding molecules, changes in extracellular metabolite composition may directly or indirectly influence membrane structure, lipid packing, and the activity of lipid transport systems [54,55,56]. In this context, circulating metabolites may act as modulators of membrane remodeling, linking tumor metabolism to alterations in cell surface lipid exposure.

### 5.1. Tumor Metabolic Microenvironment

Solid tumors often develop a unique metabolic microenvironment characterized by hypoxia, nutrient competition, and accumulation of metabolic byproducts. Among these, lactate is one of the most abundant metabolites produced by cancer cells due to enhanced glycolytic activity [24,27]. Lactate can reach high concentrations in tumor tissues and surrounding fluids, influencing immune cell behavior, angiogenesis, and extracellular matrix remodeling [24,27,44].

Beyond its role as a metabolic intermediate, lactate may also influence membrane-associated processes. Changes in extracellular pH associated with lactate accumulation can alter membrane charge distribution, protein–lipid interactions, and ion transport across the plasma membrane [24,27]. Such effects may indirectly influence lipid transporter activity or membrane lipid mobility, potentially contributing to changes in lipid asymmetry.

Other metabolic intermediates, including succinate, fumarate, and other tricarboxylic acid cycle derivatives, can accumulate in certain tumors due to mutations in metabolic enzymes [70]. These metabolites have been shown to function as signaling molecules that regulate gene expression, inflammatory responses, and oxidative stress pathways [70]. Although their direct effects on membrane lipid asymmetry remain largely unexplored, their influence on cellular signaling networks suggests possible indirect mechanisms linking metabolic state to membrane organization.

### 5.2. Lipid-Derived Metabolites and Membrane Properties

Lipid-related metabolites represent another class of molecules that may directly affect membrane structure. Tumor metabolism frequently generates increased levels of lysophospholipids, fatty acids, and acylcarnitines, which can be detected in both tumor tissues and circulation [41,60,63].

Lysophospholipids are amphipathic molecules capable of inserting into lipid bilayers and altering membrane curvature and fluidity. Their presence can affect lipid packing density and may influence the accessibility of phospholipids to lipid transport proteins [53,54]. In addition, certain lysophospholipids function as signaling molecules that regulate cell proliferation, migration, and inflammatory responses, processes closely associated with tumor progression [36,37].

Acylcarnitines, which accumulate during altered mitochondrial fatty acid oxidation, also possess amphipathic properties and may interact with membrane structures [25]. Elevated levels of circulating acylcarnitines have been reported in several cancer types and may reflect metabolic stress or mitochondrial dysfunction [39].

Furthermore, oxidized phospholipids generated during oxidative stress can significantly alter membrane properties. These oxidized lipids often exhibit increased polarity and altered packing characteristics, which may influence membrane curvature and lipid mobility [71,72].

### 5.3. Amphipathic Metabolites and Membrane Curvature

Membrane curvature and mechanical properties are strongly influenced by the composition of lipids and amphipathic molecules within the bilayer. Amphipathic metabolites can insert into membranes asymmetrically, generating curvature stress that promotes membrane remodeling events such as vesicle formation, endocytosis, and EV release [52,53].

Cancer cells are known to produce large numbers of EVs, including exosomes and microvesicles, which contribute to intercellular communication within the tumor microenvironment [45,47]. Formation of these vesicles often involves localized disruption of membrane lipid asymmetry and redistribution of phospholipids between membrane leaflets [5]. Metabolites capable of altering membrane curvature may therefore indirectly influence lipid asymmetry by facilitating membrane budding or vesicle formation processes.

In addition, amphipathic metabolites may affect membrane fluidity and phase behavior, particularly within cholesterol-rich membrane microdomains. These changes could influence the spatial organization of lipid transport proteins and signaling complexes. Beyond lipid-derived metabolites, a broader range of extracellular metabolites with amphipathic or signaling properties may also contribute to membrane remodeling. These metabolites can influence membrane curvature, lipid packing, and the activity of lipid transport systems, thereby linking the tumor metabolic environment to membrane dynamics.

### 5.4. Metabolite–Membrane Feedback Loops

Taken together, these observations support the concept that metabolites generated by cancer metabolism may participate in feedback loops that influence membrane structure and function. Changes in metabolic flux can alter the abundance of amphipathic metabolites and lipid intermediates capable of interacting with membrane structures. These molecules may modify membrane curvature, fluidity, and lipid packing, potentially affecting the activity of lipid transport proteins and the distribution of phospholipids across the membrane bilayer [53,55].

Through these mechanisms, membrane lipid asymmetry may act as a dynamic interface between cellular metabolism and extracellular signaling. Altered lipid exposure on the cell surface can influence immune recognition, receptor signaling, and cell–cell interactions within the tumor microenvironment [20]. Thus, metabolic regulation of membrane remodeling may represent an underappreciated layer of control linking tumor metabolism to cancer progression and therapeutic response.

Further investigation will be required to define the specific metabolites and molecular pathways involved in this process. Integrating metabolomics, lipidomics, and membrane biology approaches may provide important insights into how metabolic states reshape membrane architecture and cell surface signaling in cancer [38,39].

## 6. Therapeutic Targeting of Membrane Lipid Asymmetry

The abnormal exposure of inner-leaflet phospholipids on the surface of tumor cells has generated significant interest as a potential therapeutic vulnerability. Because healthy cells typically maintain strict lipid asymmetry, the presence of PS, PE, or other normally internal phospholipids on the outer leaflet of tumor cell membranes creates opportunities for selective targeting [18,19,20]. Several therapeutic strategies have been developed to exploit this property, ranging from lipid-binding antibodies to membrane-active peptides and lipid-targeting nanotherapeutics. Representative therapeutic strategies targeting membrane lipid asymmetry in cancer are summarized in Table 3.

### 6.1. Phosphatidylserine-Targeting Strategies

Among membrane lipids, PS has received the greatest attention as a therapeutic target. As discussed above, PS exposure is frequently observed on tumor cells as well as on tumor-associated endothelial cells within the tumor vasculature [19,21]. Importantly, PS exposure can contribute to an immunosuppressive tumor microenvironment by promoting anti-inflammatory signaling and suppressing immune activation [20].

To exploit this feature, several PS-targeting therapeutic agents have been developed. One approach involves monoclonal antibodies that recognize PS or PS-associated complexes on tumor cells [49]. These antibodies can promote immune-mediated destruction of tumor cells by engaging immune effector mechanisms such as antibody-dependent cellular cytotoxicity or complement activation.

Another strategy utilizes lipid-binding proteins or nanoparticles designed to bind exposed PS and deliver therapeutic cargo selectively to tumor tissues [76]. Such approaches aim to exploit the differential lipid exposure between tumor and normal cells to enhance treatment specificity.

In addition to direct targeting of tumor cells, PS-targeting agents may also modulate the tumor microenvironment. By blocking PS-mediated immunosuppressive signaling, these therapies may enhance anti-tumor immune responses and potentially synergize with immune checkpoint inhibitors [20].

### 6.2. Targeting PE and Membrane Charge

PE represents another potential membrane lipid target in cancer. Although PE is primarily located in the inner leaflet of the plasma membrane in healthy cells, tumor cells can display PE on their outer membrane surface under conditions of metabolic stress or membrane remodeling [21].

Several peptides and small molecules have been developed that preferentially bind to PE-rich membranes. Some antimicrobial peptide derivatives exhibit selective toxicity toward cancer cells by interacting with negatively charged or PE-exposing membranes [22]. These molecules can disrupt membrane integrity, induce pore formation, or trigger cell death pathways.

Because tumor cell membranes often exhibit altered lipid composition and increased negative surface charge due to exposed aminophospholipids, they may be more susceptible to membrane-active agents than normal cells. This property has inspired the development of cationic peptides and synthetic amphipathic molecules designed to selectively disrupt tumor cell membranes [22].

### 6.3. Membrane-Disrupting Peptides and Lipid-Selective Cytotoxicity

Membrane-disrupting peptides represent a class of therapeutic agents that exploit differences in membrane lipid composition between cancer cells and normal cells. Many of these peptides are derived from naturally occurring antimicrobial peptides that target microbial membranes enriched in negatively charged phospholipids [22].

Because tumor cell membranes often display increased levels of exposed PS, PE, or other anionic lipids, they can resemble microbial membranes in terms of surface charge and lipid composition. As a result, certain antimicrobial peptide derivatives have been shown to preferentially bind and disrupt cancer cell membranes [22].

These peptides typically function by inserting into lipid bilayers and forming pores or destabilizing membrane structure. The resulting membrane damage can lead to rapid cell death, often independent of traditional apoptosis pathways [22]. Such mechanisms may be particularly valuable for targeting cancer cells that have developed resistance to conventional chemotherapeutic agents.

### 6.4. Lipid-Targeted Drug Delivery Systems

Altered membrane lipid composition in cancer cells also provides opportunities for targeted drug delivery. Nanoparticle-based systems can be engineered to recognize specific lipid features on tumor cell membranes, including exposed PS or other lipid signatures [76].

For example, lipid-binding peptides or antibodies can be incorporated into nanoparticle surfaces to enhance selective binding to tumor tissues. These nanoparticles can deliver chemotherapeutic drugs, nucleic acids, or imaging agents directly to cancer cells, improving therapeutic efficacy while reducing off-target toxicity.

In addition, liposomes and other lipid-based carriers can exploit the altered membrane properties of tumor cells to enhance cellular uptake. Because membrane fluidity and lipid organization are often altered in cancer cells, these changes may influence nanoparticle–membrane interactions and facilitate drug delivery.

### 6.5. Metabolic Vulnerabilities and Membrane Targeting

An emerging concept is that metabolic reprogramming in cancer may create membrane vulnerabilities that can be exploited therapeutically. As discussed in earlier sections, metabolic stress can influence lipid transport systems, membrane lipid composition, and the exposure of inner-leaflet phospholipids on the cell surface [25,26].

Targeting these metabolically driven membrane alterations may provide new therapeutic opportunities. For example, metabolic inhibitors that perturb lipid biosynthesis or mitochondrial metabolism may increase membrane lipid exposure, thereby sensitizing tumor cells to lipid-targeting therapies [60]. Similarly, therapies that modulate oxidative stress or calcium signaling may indirectly influence membrane lipid asymmetry and enhance the effectiveness of membrane-directed agents [31,33].

These strategies highlight the potential value of integrating metabolic therapy with membrane-targeted approaches. By understanding how cancer metabolism reshapes membrane lipid organization, it may be possible to design combination therapies that exploit both metabolic and membrane vulnerabilities in tumor cells.

## 7. Future Perspectives

The concept that membrane lipid asymmetry may be influenced by cellular metabolism opens new perspectives for understanding how metabolic states shape cell surface signaling and tumor–microenvironment interactions. Although the molecular mechanisms governing lipid asymmetry have been studied for several decades, most previous work has focused on the structural and enzymatic regulation of lipid transport systems [6,7]. The emerging recognition that metabolic pathways may modulate these processes suggests that membrane asymmetry should be considered within the broader framework of cellular metabolic regulation.

One important direction for future research involves integrating lipidomics and metabolomics approaches to better understand how metabolic changes influence membrane organization. Advances in mass spectrometry-based lipid profiling have revealed substantial heterogeneity in membrane lipid composition across different cancer types [41]. Combining these approaches with metabolomic profiling may help identify metabolic signatures associated with altered membrane lipid distribution and lipid exposure patterns [38,39].

Another promising area of investigation concerns the role of the tumor microenvironment in regulating membrane lipid asymmetry. Tumor tissues are characterized by complex metabolic gradients, including hypoxia, nutrient deprivation, and accumulation of extracellular metabolites [42,44]. These conditions may create localized environments that promote membrane remodeling and lipid redistribution. Understanding how microenvironmental factors influence lipid transport systems and membrane organization may reveal new insights into tumor progression and therapeutic resistance.

The potential involvement of circulating metabolites in membrane regulation also represents an emerging field of interest. Metabolomic studies continue to identify numerous previously uncharacterized small molecules present in circulation, many of which remain functionally uncharacterized [77,78].

Technological advances in live-cell imaging and membrane biophysics are also likely to accelerate progress in this area. Super-resolution microscopy, lipid-sensitive fluorescent probes, and biophysical membrane models may enable direct visualization of lipid redistribution events in living cells. These approaches could provide valuable insights into how metabolic perturbations influence membrane dynamics and lipid transport processes in real time [53,54].

From a translational perspective, further exploration of membrane lipid asymmetry may provide new opportunities for cancer diagnosis and therapy. Because exposed phospholipids on tumor cells represent relatively selective surface markers, they may serve as targets for imaging agents, targeted drug delivery systems, or immunotherapeutic strategies [19,20]. Understanding how metabolic conditions regulate lipid exposure may therefore help identify conditions that enhance the susceptibility of tumor cells to membrane-targeted treatments.

Finally, a deeper understanding of the connections between metabolism and membrane organization may reshape how we view the plasma membrane in cancer biology. Rather than acting solely as a structural boundary, the plasma membrane may function as a metabolically responsive signaling interface that integrates intracellular metabolic state with extracellular communication. Elucidating how metabolic states regulate membrane lipid asymmetry may therefore reveal new regulatory layers linking metabolism, membrane biology, and tumor progression.

## Figures and Tables

**Table 2:** Metabolites Potentially Influencing Membrane Remodeling.

Metabolite Class	Representative Metabolites	Potential Effects on Membrane	Cancer Relevance	Key Reference
Organic acids	Lactate	Alter extracellular pH and membrane interactions	Tumor metabolic microenvironment	[24,27]
TCA intermediates	Succinate, fumarate	Influence stress signaling pathways	Oncometabolite signaling	[70]
Lipid metabolites	Lysophospholipids	Modulate membrane curvature and lipid packing	Membrane remodeling	[36,37]
Fatty acid derivatives	Acylcarnitines	Interact with membrane structures	Metabolic stress	[41,60]

**Table 3:** Therapeutic Strategies Targeting Membrane Lipid Asymmetry in Cancer.

Target Lipid or Membrane Feature	Representative Agents	Mechanism of Action	Therapeutic Strategy	Key Reference
PS	PS-targeting antibodies (e.g., bavituximab)	Bind exposed PS on tumor cells and vasculature	Immune activation	[49]
PS	Annexin-based probes	Bind PS	Imaging/targeted delivery	[22]
PE	Duramycin and related peptides	Bind to PE-rich membranes	Experimental targeting	[21]
Membrane negative charge	Cationic antimicrobial peptide derivatives	Bind to anionic tumor membranes	Membrane disruption	[22]
Tumor membrane lipid composition	Lipid-targeted nanoparticles	Enable drug delivery	Drug delivery	[76]
Tumor membrane remodeling	Amphipathic membrane-active peptides	Induce membrane destabilization and pore formation	Direct cytotoxicity	[22]

## Data Availability

No new data were created or analyzed in this study. Data sharing is not applicable to this article.

## Abbreviations

EV	Extracellular vesicle
PC	Phosphatidylcholine
PE	Phosphatidylethanolamine
PI	Phosphatidylinositol
PS	Phosphatidylserine
ROS	Reactive oxygen species
